# Corporations’ use and misuse of evidence to influence health policy: a case study of sugar-sweetened beverage taxation

**DOI:** 10.1186/s12992-019-0495-5

**Published:** 2019-09-25

**Authors:** Gary Jonas Fooks, Simon Williams, Graham Box, Gary Sacks

**Affiliations:** 1 School of Humanities and Social Sciences, Aston University, Birmingham, B4 7ET UK; 2 School of Law, University of Reading, Reading, Berkshire RG6 6AH UK; 3 WHO Collaborating Centre for Obesity Prevention, Deakin University, Melbourne, Victoria 3125 Australia

**Keywords:** Commercial determinants of health, Agnotology, Corporate misuse of science, Corporate political activity, Sugar tax, Corporate misuse of evidence

## Abstract

**Background:**

Sugar sweetened beverages (SSB) are a major source of sugar in the diet. Although trends in consumption vary across regions, in many countries, particularly LMICs, their consumption continues to increase. In response, a growing number of governments have introduced a tax on SSBs. SSB manufacturers have opposed such taxes, disputing the role that SSBs play in diet-related diseases and the effectiveness of SSB taxation, and alleging major economic impacts. Given the importance of evidence to effective regulation of products harmful to human health, we scrutinised industry submissions to the South African government’s consultation on a proposed SSB tax and examined their use of evidence.

**Results:**

Corporate submissions were underpinned by several strategies involving the misrepresentation of evidence. First, references were used in a misleading way, providing false support for key claims. Second, raw data, which represented a pliable, alternative evidence base to peer reviewed studies, was misused to dispute both the premise of targeting sugar for special attention and the impact of SSB taxes on SSB consumption. Third, purposively selected evidence was used in conjunction with other techniques, such as selective quoting from studies and omitting important qualifying information, to promote an alternative evidential narrative to that supported by the weight of peer-reviewed research. Fourth, a range of mutually enforcing techniques that inflated the effects of SSB taxation on jobs, public revenue generation, and gross domestic product, was used to exaggerate the economic impact of the tax. This “hyperbolic accounting” included rounding up figures in original sources, double counting, and skipping steps in economic modelling.

**Conclusions:**

Our research raises fundamental questions concerning the bona fides of industry information in the context of government efforts to combat diet-related diseases. The beverage industry’s claims against SSB taxation rest on a complex interplay of techniques, that appear to be grounded in evidence, but which do not observe widely accepted approaches to the use of either scientific or economic evidence. These techniques are similar, but not identical, to those used by tobacco companies and highlight the problems of introducing evidence-based policies aimed at managing the market environment for unhealthful commodities.

**Electronic supplementary material:**

The online version of this article (10.1186/s12992-019-0495-5) contains supplementary material, which is available to authorized users.

## Background

Sugar sweetened beverages (SSB) are a major source of sugar in the diet. Although trends in consumption vary across regions, in many countries, particularly LMICs, their consumption continues to increase [1]. In response, a growing number of governments have introduced a tax on SSBs as part of broader programmes aimed at reducing sugar consumption [2]. Proposed policies are typically proceeded by public consultations, the increasing use of which reflects a global process of reform that draws heavily on US administrative law and its cost-benefit approach to regulatory review and policy formation [3]. This paper takes a case study-approach to examining corporate actors’ use of evidence in written consultation submissions to the South African National Treasury’s proposed tax on sugar sweetened beverages (SSB). Specifically, it explores corporations’ use of agnogenic practices to shape policy actors’ understanding of the policy and its effects.

By agnogenic practices we refer to methods of representing, communicating, and producing scientific research and evidence which work to create ignorance or doubt irrespective of the strength of the underlying evidence [4]. Agnogenic methods of research representation and communication by corporations vary considerably, ranging from discursive practices that demand impossibly high standards of scientific proof [5, 6] to withholding clinical trial data [7]. Agnogenic practices relevant to producing scientific research and evidence are equally diverse and include devising research protocols that are more likely to produce desired results [4, 8–10] or simply ensuring that some research is not undertaken in the first place for fear of producing unfavourable results. There is now a wealth of evidence examining the role that corporate actors play in agnogenesis (the production of information or ideas that create ignorance or doubt beyond that merited by empirical evidence) [6]. This has primarily centred on corporate influence in primary scientific research [10–19], systematic reviews [16, 18, 20–22], and science communication [12, 14, 18, 23–26]. And whilst there is an emerging body of work on the production of ignorance in the context of state regulatory agencies [27, 28], agnogenic behaviour by corporate actors in presenting evidence within policy-making processes is relatively underexplored despite strong business dominance in the processes used to collect evidence which underpin evidence informed policy [29].

In addition, much of the existing literature examining the interface between corporations and policy- relevant science either simply describes industry influence on science and its communication or demonstrates its effects [10, 23, 30–33], rather than model the discrete techniques corporate actors use to shape how science and knowledge are understood. There are some notable exceptions to this [5, 34–39]. However, different methodological approaches and ontological perspectives taken within this limited literature have produced what are effectively insular studies that do not share a common conceptual vocabulary, which is likely to impede the cross-fertilisation of ideas between scholars working within different policy contexts. Moreover, existing studies tend to examine agnogenic practices independently of one another without exploring how they are combined to support evidence claims. They also tend to ignore industry claims relating to economic impacts. Both of these factors are key to helping policy actors understand how agnogenesis takes effect and evaluate corporate claims appropriately. Consequently, we build on existing conceptual frameworks of corporate agnogenesis [5, 34] and develop a synergic, stratified model of industry misuse of evidence, which takes account of how interdependencies between different techniques shape evidence-based narratives within corporate submissions. By focusing on South Africa, we also address the relative dearth of research on corporations’ use of evidence in health policy in low-and-middle income countries. After providing a brief overview of the key claims made within corporate actors’ submissions and the extent to which claims are nominally supported by evidence, we outline techniques of agnogenesis, indicating how they interact and support one another. This is followed by a brief section providing a more detailed explanation of how techniques interlink with and reinforce one another. In the discussion, we examine the relevance of these practices for appraising the merits of involving corporations in health policy-making.

Our selection of SSB taxation as a case study is based on three observations. First, efforts by governments internationally to use fiscal levers as a means of addressing rising levels of type 2 diabetes, obesity, and associated cardiovascular disease have met with fierce industry opposition [40, 41]. This opposition is consistent with (and potentially prompted by) strong evidence that SSB taxation reduces SSB consumption [42, 43], some evidence that it may also drive reductions in sales of diet drinks [44–46], and emerging findings that some substitution effects may be captured by other market actors (as in the case of milk or coffee) or are not so readily commodified (as in the case of water) [47, 48]. That this combination of effects is likely to reduce sales and corporate earnings significantly increases the incentives for corporate actors to engage in what Parkhurst has termed “strategic technical bias” (questionable uses of evidence that depart from scientific best practice) [35, 36, 49]. Second, evidence linking SSB consumption to obesity and elevated risk of metabolic and cardiovascular diseases is voluminous, growing, and methodologically diverse [50–52]. Combined with the fact that diet-related diseases have complex aetiologies, and that, historically, evidence linking SSB taxes to weight loss has been mixed [53–55], this increases the opportunities for corporate actors to engage in strategic technical bias. Third, evidence of the economic, substitution, and complementarity effects of SSB taxation is emerging rather than settled [47], which raises additional sources of uncertainty and, therefore, opportunities for technical biases. The study represents the first systematic, critical examination of policy-facing research communication by corporations beyond the UK and presents the first synergic model of corporate agnogenesis.

## Method

### Data collation

A desk based approach was taken to collating written responses to the South African National Treasury’s policy paper on a taxation of sugar sweetened beverages [56, 57] by corporate actors (hereafter industry submissions), which we defined to include: companies in the food and drink sector and SSB supply chain and the business associations that represent them; professional service firms with food and drink sector clients and the professional associations that represent them. Submissions are not collated in a single publicly accessible website and so were obtained through a mix of requests to public officials involved in the consultations, Google searches of respondents’ web-sites (search string - “respondent’s site”:(url) “sugar levy” OR “sugar tax” OR “industry levy” OR [tax AND “sugar sweetened beverages”] OR [tax AND “soft drinks”] OR [levy AND “sugar sweetened beverages”] OR [levy AND “soft drinks”]) and email requests to respondents. Of the five submissions obtained via this process, two were excluded for in-depth review as they did not cite evidence. This left three submissions for close analysis, those of: the American Chamber of Commerce South Africa (AmCham SA), the Beverage Association of South Africa (BEVSA), the peak industry association for South African based SSB manufacturers, and Coca-Cola, the company which arguably stood to lose most from the tax. In addition, we undertook an in-depth critical appraisal of an industry commissioned report by Oxford Economics [58], which was cited at length in BEVSA’s and Coca-Cola’s submission.

We used several methods to collate evidence cited in the submissions and Oxford Economics report. Peer-reviewed research was identified via Web of Science and PubMed Central. Searches of authors’ institutional web-sites were used to identify research consultant, company, and (non-peer reviewed) academic reports. Where this proved unsuccessful, we performed general internet searches using the search engine Google and requested copies from authors via email. The same protocol was used where the cited (primary) source was not the ultimate (secondary) source of the evidence claimed in the submission reviewed (see Results). Peer-reviewed research articles on the effects of SSB taxation on consumer behaviour based on calculations of cross-price elasticities were collated to strengthen our evaluation of Oxford Economics’ modelling. These were identified using relevant search terms via Web of Science and PubMed Central and by hand searches of reference lists of studies identified as relevant (see Additional file 1: Table S1). Finally, we contacted (via-email) authors of studies and reports cited in evidence and analysed in depth (*n* = 3) to seek clarification of specific points, but received no replies.

### Data analysis

We undertook three analyses. First, we performed a source analysis of industry submissions, which involved: identifying policy relevant propositions within submissions (executive summaries and introductory sections were excluded); assessing whether propositions were substantiated with reference to an ostensibly validating source; classifying the type (e.g. method of funding and publication) and availability of the source. We defined “relevant propositions” as statements or assertions that expressed an anticipated effect of, or judgement of fact supportive of an anticipated effect of, the policy beyond the intended direct effects of SSB taxation (encouraging a substantive decline in consumption of SSBs), but excluding assertions relating to the industry’s pre-levy contribution to the economy, such as employment associated with the non-alcoholic beverage industry. Evidence availability was examined by transposing the search strategies used to collate the evidence (outlined above) into thematic codes. Where evidence was obtained directly from authors we used a web-archiving tool (https://archive.org/web) to determine availability at both the time of publication and immediately after the deadline for consultation submissions. A sub-sample (10%) of the results of this analysis was coded by SW. Disagreements over differences in coding were resolved through discussion and consensus.

The second method of analysis combined a verification-oriented cross-documentary analysis with an interpretative analysis used to identify conceptual themes and explore interconnections between different techniques. This used a backward mapping strategy to compare references made to evidence (where cited) with their supporting sources to examine how they had been used. Where the supporting (primary) source was not the original source of evidence for the proposition, we applied the same approach to the underlying (secondary) source. The results of this process were thematically analysed (by GF) using the techniques of constructivist grounded theory [59, 60]: systematic conceptual coding (using Nvivo software); constant comparison; discourse sensitivity; attention to divergent data; conceptual conclusions. A hybrid approach (part inductive and emergent and part deductive) [61] was taken to coding. To this end, our analysis was informed by four literatures: social constructivist perspectives of science [62, 63], which work on the premise that facts are socially and interactionally constructed and open to alternative interpretations; studies on the (mis) use of science by corporations [5, 12, 64]; the literature on logical fallacies [65]; and studies on evidence synthesis and weight of evidence analyses [66, 67]. The micro (first level) themes (described as techniques in the analysis) were grouped under broader categories (which we describe as practices) and (where relevant) synthesised with conceptual categories used in the existing literature [5]. Emerging ideas were discussed by the wider team at interim analytic meetings. A sub-sample of the material (10%) was coded by two other researchers (SW, GB). Disagreements over differences in coding were resolved through discussion and consensus.

Third, the critical appraisal of Oxford Economics’ report [58] was undertaken by evaluating assumptions, data sources, information uncertainties, and unquantified/quantified costs and benefits within economic models using the backward mapping approach outlined above [68]. The results of this analysis were used to develop the interpretive analysis.

## Results

### Overview of submissions

Industry submissions set out a metanarrative of “policy dystopia” [69]. Predicated on accumulating anticipated “social bads”, this stressed the policy would cause widely dispersed adverse social and economic consequences and fail on its own (public health) terms. Among other things, corporate actors claimed that the tax would: trigger tens of thousands of job losses concentrated in small-scale farms and spazas (informal convenience stores usually run from home) and reduce employment growth; exacerbate the broader fiscal and societal costs associated with unemployment (by, for example, reducing the overall tax take); damage the competitiveness of the non-alcoholic beverage industry; undermine South Africa’s National Development Plan (specifically its aim to increase economic growth, eliminate poverty, and increase employment); trigger business failures across the supply chain; lead to reduced revenue for farmers; dissuade international investors from investing in South Africa; increase the risk of a credit downgrade; disproportionately fall on lower-income households; and have a negligible impact on population health. Claims that SSB would not measurably improve health outcomes were based on three supporting propositions: first, because SSBs constituted a small proportion (3%) of energy intake in South Africa any decline in SSB consumption was unlikely to significantly reduce obesity; second, consumption of sugar within South Africa was declining and, therefore, not a key driver of the country’s increasing obesity rate; and, third that consumers would simply substitute SSB consumption with other energy-dense products [70–72].

**Fig. 1:**
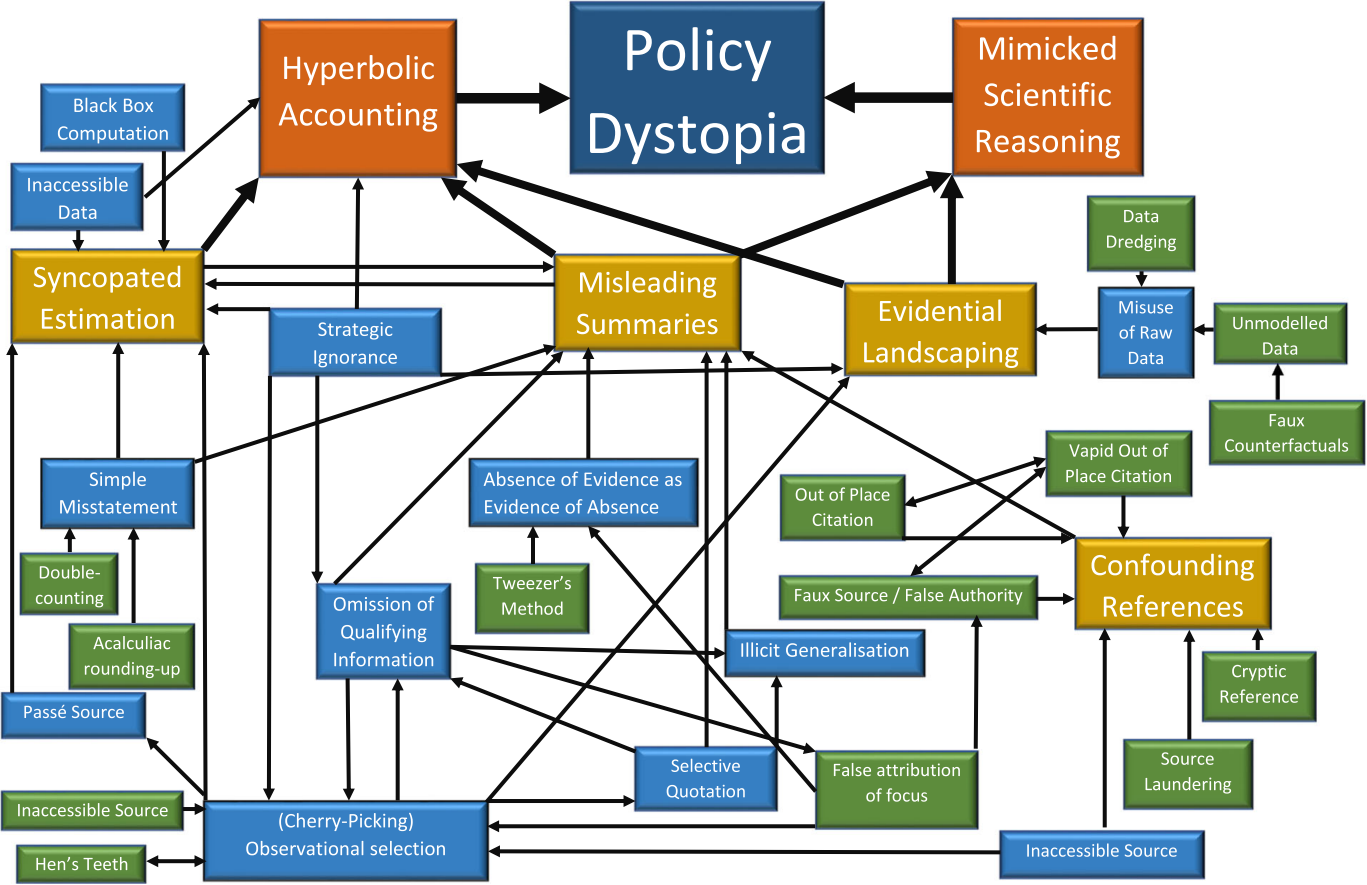
Model of Corporate Agnogenesis of Soft Drink Companies in the context of South Africa’s Consultation on a Proposed Taxation on Sugar-Sweetened Beverages

Summarised explanations of the agnogenic practices and techniques used to support this dystopic narrative are outlined in Table 1 immediately below. Where techniques work to similar effects (e.g. *false attribution of focus* and *selective quotation*) or are linked by a common theme (e.g. *cryptic references* and *faux sources*) we group them together under related practices (i.e. *misleading summaries and* and *confounding references.*). We go on to discuss our results under two meta-practices: *mimicked scientific reasoning* and *hyperbolic accounting.* We use the term *mimicked scientific reasoning* to describe practices and techniques that misrepresent, and work to circumvent, the weight of evidence concerning the effects of SSBs and SSB taxes on obesity and diet-related diseases, including, for instance, misrepresenting the focus and objectives of studies and omitting important qualifying information. Scientific reasoning is *mimicked* in the sense that evidence use and appraisal appears, on the face of it, to take an unprejudiced, evidence-informed assessment of the relevant science. In practice, however, the approach fails to observe accepted principles of deductive and inductive reasoning, does not observe accepted conventions associated with how to accurately support evidence-based claims, and does not appropriately take into account weight or strength of evidence approaches to evidence appraisal. *Hyperbolic Accounting*, by comparison, encompasses techniques and practices that exaggerate the stated economic impact of proposed policies (on employment, public revenue generation, and gross domestic product), such as failing to fully articulate key steps in economic modelling (*syncopated estimation*) or counting economic impacts more than once (*double counting*). Although we deal with these meta-practices separately for ease of understanding, in practice, agnogenic techniques cut across efforts to misrepresent the weight of evidence concerning the effects of SSBs and SSB taxes on obesity and diet-related diseases and exaggerate the economic impacts of SSB taxes (see Fig. 1).

**Table 1:** Agnogenic practices and techniques by soft drink manufacturers in the consultation on South Africa’s proposed sugar-sweetened beverages policy

Practices	Techniques	Description
Confounding Referencing		• The misleading use of references which either overstates or gives an entirely false impression of support for a claim or obstructs evidence appraisal.
	Cryptic references	• An opaque reference that provides insufficient information to easily locate the original source and which serves to obstruct evidence appraisal.
	Faux sources / False authority	• A *faux source* involves providing an incorrect source for key data. The concept overlaps with an appeal to a *false authority*, where an alleged authority is used as evidence to support a claim, which, in fact, is not an authority on the facts relevant to the claim.
	Out-of-place citations	• References that give a false impression of support for a proposition as a result of being misplaced in the text. These take various forms and can be used to validate illicit generalisations or simply provide a faux source for a key proposition.
	Vapid out-of-place citations	• A hybrid confounding reference (combining an out-of-place citation and a faux source) which contains relatively useless contextual information that fails to support, and has no direct relevance, to the claim in the text.
	Source laundering	• Provision of a relatively independent source which obscures the use of industry data as the underlying support for the proposition.
	.....	.....
	Inaccessible source	• The use of a source that is not publicly available.
Misleading Summaries		• Inaccurate reporting of objectives, findings, and conclusions of sources.
	Absence of evidence as evidence of absence	• A logical fallacy aimed at representing a relationship that has not been satisfactorily explored as evidence that no relationship exists (usually used in combination with other techniques, such as omission of qualifying information).
	False attribution of focus	• Misrepresentation of the focus of studies.
	Omission of important qualifying information	• A specific variant of strategic ignorance characterised by precise but inaccurate reporting of study findings in which important qualifying information that significantly changes the implications of the findings is omitted.
	Selective quotation	• Reporting extracts either out of context or by omitting qualifying information to give a misleading impression of either the study quoted or the research upon which it is based.
	Simple misstatement of key/study findings	• Erroneously and unambiguously claiming that a study has produced a specific finding.
	‘The Tweezers Method’	• The practice of picking phrases out of context from peer-reviewed studies with the effect of changing the emphasis and/or intended meaning of the original text.
	Acalculiac rounding-up	• Rounding-up estimates without cause or explanation.
	Double-counting	• Counting an economic impact (or part of an impact) more than once.
	Illicit Generalisation	• A logical fallacy where the underlying evidence is insufficiently developed to support an inductive generalisation.
Evidential Landscaping		• Either promoting alternative evidence (a parallel evidence base) to shift the evidential basis upon which the policy is being discussed and evaluated or purposefully excluding relevant evidence
	Data dredging (misuse of raw data)	• Presenting and/or analysing data to depict relationships or trends that either misrepresent actual relationships or obscure other contradictory relationships and/or trends in the data.
	Unmodelled data (misuse of raw data)	• Homespun trend analysis summarising patterns across time that ignores key confounding variables or pre-existing/underlying trends. In this latter sense, unmodelled data may involve a *faux counterfactual*, where the impact of an intervention is not appropriately explored by comparing the world in which the intervention occurred with the world in which it did not.
	Observational Selection/Cherry-Picking	• The practice of highlighting individual studies or data to support a pre-determined conclusion, whilst ignoring contradictory (and typically stronger) evidence.
	The ‘Hens’ teeth’ technique	• An egregious form of cherry-picking that involves foregrounding obscure, outlying studies.
	Passé Source	• Cherry-picking an older source to support an assumption, which although fairly reflecting the state of scientific knowledge when published has since been superseded by developments in the evidence-base.
	Strategic ignorance	• The technique of ignoring findings and evidence-backed observations in cited sources that contradict unsupported or weakly supported claims.
Syncopated Estimation		• Missing or failing to fully articulate key steps in economic modelling (including, but not limited to, the failure to: provide a range of estimates to reflect uncertainties in assumptions; fairly review the literature relevant to specifying assumptions; provide a clear and comprehensive assessment of assumptions).
	Black-box Computation (information asymmetries)	• Opaque, unverifiable steps in economic modelling.
	Inaccessible Data (information asymmetries)	• The reliance on privately held data in economic assessments.

### Mimicked scientific reasoning

#### Confounding referencing

Industry submissions took a misleading approach to referencing sources. Techniques such as *source laundering* (providing secondary sources to mask the use of industry data) and *faux sources* (falsely attributing data to a cited source, AmCham SA only) gave a misleading impression of the breadth of sources and evidence supporting claims (see also *out-of-place citations*, discussed under *Misleading Summaries* below).

*Source laundering* (Table 1) AmCham SA referenced a 2013 report jointly produced by Oxford Economics and the International Tax and Investment Center (hereafter the 2013 Oxford Economics report) [73] to support the claim that Denmark had abolished their “fat … and sugar tax”, in part, due to cross-border shopping [71]. Oxford Economics’ comments on cross-border shopping in Denmark drew exclusively from a Danish Food and Drink Federation (DI Fødevarer) factsheet on the tax on saturated fat in Denmark [74]; and a EURACTIV report of a survey by the Danish Grocers’ Trade Organisation [75] (also see *Information Asymmetries* below). By creating an additional step in the process of assessing the methodology of original sources and verifying that their findings have been accurately reported, the technique complicated the process of evidence appraisal. In the present case, Oxford Economics’ 2013 report had cited the DI Fødevarer factsheet to support the contention that a Danish family could save at least US$455 (EUR350) a year by shopping in Germany, despite the factsheet containing no such claim (see *faux source* below and Table 1) [74]. In addition, Oxford Economics had noted that the change in shopping habits outlined in the EURACTIV report related to “beverages”; the natural implication being that this applied exclusively to non-alcoholic beverages [73]. In fact, the EURACTIV report noted clearly that the survey results referred to purchasing behaviour for soft drinks *and* beer combined and that the Danish government had introduced higher taxes on beer (amongst other things) in the January of the year the survey was conducted (which, all things being equal, was likely to have increased the demand for cross-border shopping in respect of alcoholic beverages) [75].

*Faux Sources* (Table 1) The importance of transparency in citing sources is underlined where laundering involves *faux sources* (falsely attributing data to a cited source) (see also *Cryptic References* below)*.* AmCham SA reported that SSB taxation was “blamed for the loss of 1,300 jobs as Danish shoppers migrated to purchasing their preferred soft drinks in Germany and Sweden” [71] citing the 2013 Oxford Economics’ report [73] in support. In practice, the report did not give a figure for job losses and only noted the “detrimental impact of introducing an SFBT on jobs and investment, its influence on transborder purchasing, alongside the administrative costs it imposes on companies” [73]. However, an almost identically worded claim relating to Denmark’s saturated fat excise duty was made in an article in *The Spectator* magazine [76] which was also cited by AmCham SA. A supporting reference is not provided in the article. However, it appears to draw on a discussion piece on Denmark’s tax on saturated fat published by Christopher Snowdon of the Institute of Economic Affairs (UK) [77], an occasional contributor to the magazine, which makes an identical claim citing a commentary in a Danish online newspaper, written by the head of Dansk Erhverv (the Danish Chamber of Commerce) and managing director of Landbrug & Fødevarer (the Danish Agricultural & Food Council). The commentary simply states, without reference to a data source or method of calculation, that, “according to our calculations, the *fat tax alone* has cost 1,300 jobs” (*emphasis added*) [78].

#### Misleading summaries

Submissions used several techniques that centred on inaccurately reporting objectives, findings, and conclusions of sources. These ranged from relatively simple cases of *misstating key findings* to *omitting important qualifying information* and the *tweezers method* [5] of picking phrases out of context, thereby changing the emphasis and intended meaning of the original text. The effect of these techniques was to transform evidence that contradicted, weakly supported or provided no support for the industry’s case into evidence that was stated to be strongly and unambiguously supportive.

One technique, used relatively heavily by AmCham SA, involved the simple *misstatement of key findings*. AmCham SA, for instance, noted that, “negative externalities and increased administrative costs, job losses, higher food prices, lower profitability for firms … were found in a study of Finland, France, the Netherlands and Hungary on food taxes” [71] led by Ecorys, a European based research and consultancy firm (hereafter Ecorys’ study or Ecorys’ report) [79]. The only explicit reference to negative externalities in Ecorys’ report related to consumer externalities (i.e. the costs to *society* not already factored into the price of the taxed products) that the taxes were designed to address [79]. On job losses Ecorys reported increases in employment in the year following the first tax increase on confectionery and chocolate in Denmark and Finland, no change in trend after the introduction of France’s tax on regular cola, and an end to employment growth following Finland’s tax increase on soft drinks. Only in the case of Hungary was the trend data consistent with AmCham’s claim. Even here, however, the report noted that employment increased following the introduction of taxes on SSB and energy drinks (but declined in the year following increases in the tax) [79] (see also under *Hyperbolic Accounting*).

Not all inaccurate reporting was so flagrant. In other cases, study results were accurately reported, but important qualifying information was omitted (*omission of qualifying information*). BEVSA (and Coca-Cola), for example, reported that, “even in Mexico, the SSB tax only reduced daily consumption of soft drinks by 17 kJ (4 Calories) per day– less than 0.2% of daily energy intake”, citing a study using Mexican sales data by Colchero, et al [42]. This accurately reflected their finding that purchases of taxed SSBs decreased by an average of 6% (− 12 mL/capita/day) in 2014. However, BEVSA (and Coca-Cola) failed to add that Colchero et al had found that decreases had grown progressively through 2014 as the tax took effect, reaching a 12% decline by December 2014 compared to pre-tax trends, even though this was reported prominently in the paper’s abstract.

In some cases, the omission of qualifying information was key to misrepresenting the focus and objectives of studies (*false attribution of focus*), which in its weaker form provided a platform to present *absence of evidence as evidence of absence*. AmCham SA, for instance, reported that the Ecorys’ study had found “no discernible improvement to public health” [71]. The study’s objective, outlined in the introduction to the report, was “to conduct a detailed analysis of the impact of food taxes on competitiveness in the agri-food sector” [79]. Although one of the questions explored by Ecorys involved, “what qualitative and quantitative results support a public health or fiscal objective”, the report noted that the study had “not focused on public health implications as a primary objective” [79]. Consequently, Ecorys gave little attention to health effects in their report, which were examined by way of a brief, unstructured review of public health research. On the back of this review, Ecorys observed that the extent to which food taxes lead to improvements in health was “still widely debated” and that “evidence from academic literature [was] still inconclusive and sometimes contradictory” [79]. It went on to report that, “the key reasons for the diversity in results of studies are the uncertainties around product substitution and the calculation methods used to translate consumption changes into particular health effects” and concluded that these issues could only be explored in depth once longer term health data had become available [79]. In its stronger form, *false attribution of focus* involved mobilising what was essentially a *faux source* to the same effect. AmCham SA, for instance, cited the 2013 Oxford Economics report [73] to support the claim that the impact of “SSBs on health outcomes is uncertain and unproven” [71], when the report did not examine the relationship between SSBs and health.

*Out-of-place citations*, a form of *confounding referencing*, gave a false impression of support for a key proposition (or propositions) because of how they were placed in the text. They were one of three techniques used to side-step the literature on substitution effects (see also *cherry picking to support illicit generalisations* and *strategic ignorance*), which although not entirely consistent, and still developing [47], indicated that switching to other products in response to increased SSB prices would only marginally offset energy reductions achieved through decreased SSB consumption [42, 44, 46, 48, 80–87].

*Vapid out-of-place* citations involved providing a reference next to major claims concerning substitution effects that only contained contextual information, and not the substantiating evidence its position in the text implied. In the first extract from AmCham SA’s submission outlined in Table 2 (A1) [71], for example, the source (#3) refers to the 2014 edition of PricewaterhouseCoopers’ Worldwide Tax Summaries [88]. The natural inference of the reference given its position immediately after “Denmark” was that it constituted evidence of the substitution effects alleged. In practice, however, PricewaterhouseCoopers’ Worldwide Tax Summaries provide basic details about tax systems for countries worldwide: as such, the reference constituted a *faux source* that simply provided descriptive information about the tax, which we summarise under A2 in Table 2.

**Table 2:** Out-of-Place Citations

**A1) Text from submission of American Chamber of Commerce in South Africa** [71] Consumers could substitute their soft drink choices with cheaper products and this behavioural change may undermine the impact of a sugar tax in terms of both health and revenue objectives. This was proven in Hungary and Denmark [3] when consumers made the following choices once a sugar tax was introduced which made their soft drink of choice too expensive: • They purchased and consumed lower-cost versions of the same product; • They purchased untaxed products with similar nutritional characteristics thereby preventing the goal of obesity reduction being reached; and • They purchased the same item from somewhere cheaper often resorting to trans-border purchasing which resulted in a lack of related revenue to that country’s fiscus. **A2) Text from PricewaterhouseCoopers’ 2014 Worldwide Tax Summaries cited as footnote 3 immediately above** [88] The first domestic distributor of certain products, as well as the acquirer of goods that are brought from abroad and used for the domestic manufacture of own products that will be sold in Hungary, are liable to pay a product tax. The duty rates from 1 January 2014 are as follows: [*text goes on to list commodities that attract the tax*] **B) Text from submission of Coca-Cola** [70] “Moreover, consumers typically substitute SSBs with other Calorie dense products, such as alcohol [20].”	

*Out-of-place* citations were also used to validate *illicit generalisations* (see Table 1). In the present case this involved providing a reference for an evidentially weak exemplification of a general claim that consumers would switch to other energy dense products. This is illustrated in the second extract (B) in Table 2 taken from Coca-Cola’s submission (also reproduced in BEVSA’s submission). Rather than review the evidence on substitution effects or cite a source to this effect, Coca-Cola simply provided a reference (#20) to support the exemplification. This appears to refer to an unpublished conference presentation by Hanks et al 2012 [89], which, in August 2016 (the completion date of the submission), was only one of four studies that had considered alcohol as a substitute and the only one to have found a positive association [45, 82, 90, 91] (see the *Hen’s Teeth* technique below). Moreover, a summary of a subsequent version of the presentation, published in *The Journal of Nutrition Education and Behavior*, provided no indication of the proportion of, or extent to which, consumers substituted to alcohol [91].

The *tweezers method* [5] was also used to conflate absence of evidence with evidence of absence. For example, Coca-Cola reported that a recent review by Malik and Hu (2015) had “concluded that there is limited evidence that consumers do not reduce their Calorie intake to offset Calories consumed in liquid form” [70]. These comments were designed to take issue with claims that SSBs have lower satiety and that consumers do not entirely offset liquid calories by reducing energy intake fully at subsequent meals [92]. The natural inference to be drawn from them was that studies exploring this potential effect had found limited evidence of its existence, when, in fact, Malik and Hu had used “limited evidence” to highlight the dearth of studies on the issue and concluded that the findings of this limited evidence-base were consistent with the argument that sugar or high fructose corn syrup (used to sweeten SSBs in the US) in liquid beverages may not suppress intake of solid foods to the level needed to maintain energy balance [93].

#### Evidential landscaping

In the most general sense *evidential landscaping* involves changing the evidential landscape upon which a policy is being discussed and evaluated. Ulucanlar et al [5] use the concept to encompass both the promotion of different types of evidence (a parallel evidence base) and the process of purposefully excluding relevant data or research. We take the first, positive, limb of evidential landscaping to describe the mobilisation of qualitatively different types of evidence or data to that driving the thinking of science on a contested issue and split the exclusion of evidence into two parts: cherry-picking (or observational selection), which has the effect of ignoring (excluding) evidence which does not support a pre-determined conclusion (see below and Table 1); and *strategic ignorance*, which describes the practice of over-looking findings and evidence-backed observations in cited sources (see below and Table 1).

##### Misuse of raw data

Submissions drew heavily on raw data, which presented a pliable, alternative evidence base (see Table 1) to peer reviewed studies and systematic reviews that broadly suggested a positive correlation between SSBs, obesity and disease [52, 93] and a negative correlation between SSB taxation and weight gain/obesity [94]. One approach involved disputing the premise of targeting sugar for special attention by focusing on trends in sugar consumption relative to other foods. Both Coca-Cola and BEVSA argued that SBBs constituted just 3% of average daily energy intake against a backdrop of declining consumption of added sugar: 46 kcal between 1991 and 2011. Increases in other energy dense foods, such as vegetable oils (105 kcal) and cereals (51 kcal), were claimed to account for the rise in average daily energy intake (191 kcal) over the period. To support the point each submission simply cited the “Food and Agriculture Organization of the United Nations” (FAO) [70, 72]. Notwithstanding the *cryptic* nature of the reference (Table 1), the statement appears to draw on FAO Food Balance sheets, which do indeed report that per capita daily supply of sugar (raw equivalent) declined from 346 kcal in 1991 to 300 kcal in 2011 (Table 3). However, a closer inspection of the data indicates that it had been *dredged* to fit the narrative [95, 96] (see Table 1). In the 23-year period preceding 2013, the most recent year FAO data had been available to Coca-Cola, per capita daily supply of sugar for 1991 is the highest reported by the FAO (see Table 4). 2011 (8th lowest reported) appears to have been taken as a cut-off because of the relatively steep-rise in reported sugar supply thereafter (2013 is the joint 5th highest). The effect of this can be illustrated by focusing on the 20-year (1994–2013) and 10-year (2004–2013) periods up to and including 2013. In the first scenario, FAO data indicate that sugar supply has still increased, but by just 17 kcal (decreases of 89 kcal and 40 kcal for vegetable oils and cereals respectively). However, in the second scenario, FAO data indicate that sugar supply has, in fact, increased by 38 kcal (decreases of 18 kcal and 47 kcal for vegetable oils and cereals respectively) (see Table 3).

**Table 3:** Food and Agriculture Organization Balance Sheets (Food Supply, Select Items)

Year	Sugar (Raw Equivalent) kcal/capita/day	Vegetable Oils (Raw Equivalent) kcal/capita/day^a^	Cereals kcal/capita/day^b^
1991	346	228	1495
1992	336	222	1498
1993	330	217	1592
1994	327	248	1549
1995	319	261	1526
1996	317	257	1526
1997	318	283	1503
1998	317	305	1556
1999	314	285	1547
2000	309	276	1594
2001	303	315	1595
2002	296	348	1579
2003	305	344	1573
2004	281	329	1585
2005	279	335	1590
2006	279	324	1538
2007	279	299	1529
2008	269	319	1493
2009	271	357	1481
2010	301	360	1532
2011	300	332	1546
2012	307	328	1527
2013	319	311	1538

^a^Oil crops (other), groundnut oil, sunflower oil, cottonseed oil, palm kernel oil,

^b^Wheat and products, rice (milled equivalent), barley and products, maize and products, rye and products, oats, millet and products, sorghum and products, cereals (other)

**Table 4:** Cherry-Picking (Observational Selection)

**A1) Text from Coca-Cola South Africa** [70] Several studies of observed market outcomes from SSB taxes in the US have found no impact on obesity rates. These studies conclude that “any reduction in soft drink consumption has been offset by the consumption of other Calories” [97] .” Their findings “cast serious doubt on the assumptions that proponents of large soda taxes make on its likely impacts on population weight [98].” **A2) Text from Fletcher, et al, 2010** [97] “Despite this evidence against the effectiveness of soft drink taxes to reduce obesity, we believe that there are at least two directions for further inquiry in this area. First, although there is no evidence that soft drink taxes improve weight outcomes in children and adolescents, the fact that children and adolescents substitute more nutritious whole milk for soft drinks when taxed suggests that there may be broader health benefits that are not yet understood. Second, most historical tax rates are considerably lower than those that have been recently proposed, so that extrapolating our results to much larger increases in tax rates may not be appropriate.”	

Another approach involved using *unmodelled data* and a *faux counterfactual* to question the effect of SSB taxation on purchases. AmCham SA, for instance, focused on the gross revenue generated by Mexico’s SSB tax, noting that, “the tax [had] delivered 50% more revenue in 2014 than budgeted” and that “it further increased in 2015 as sugar sweetened soft drinks grew in volume (which indicates a bounce back from consumption decrease)” [71]. The underlying claim contradicts peer-reviewed studies exploring the impact of the tax which arrive at the opposite conclusion, partly by taking per capita measurements and adjusting for macroeconomic variables that affect beverage purchases over time, but also by selecting a logical counterfactual and focusing on changes in sales relative to trend (i.e. comparing volumes of taxed purchases following the introduction of tax with estimated volumes that would have been purchased based on pre-tax trends) [42, 43].

#### Observational selection (cherry-picking)

The concept of *cherry picking* (or, more formally, observational selection) is widely used in the literature as a blanket term to describe a broad range of practices in which individual studies or data are highlighted to support a pre-determined conclusion, whilst contradictory (and typically stronger) evidence is ignored [11, 35, 36, 99–101]. One advantage of this undifferentiated use of the term is that it highlights the pervasiveness of the practice. However, it can overlook considerable inventiveness in evidence selection and obscure how the practice is combined with other agnogenic techniques (see also the discussion of *false attribution of focus* above), which is key to their agnogenic potential.

The conference presentation by Hanks, et al (2013) cited by BEVSA and Coca-Cola (see *Out-of-Place* citations above), for instance, represents an example of the *hen’s teeth* technique: an obscure, outlying study cited to support industry claims-making around substitution effects (see Table 1). Moreover, it also exemplifies the close relationship between *cherry picking* and *illicit generalisations*: Coca-Cola (and BEVSA) having made a population level claim suggesting that consumers substitute to other calorie dense products on the back of a focused, underpowered study. Equally, AmCham SA’s use of the 2013 Oxford Economics report [73] to question the evidence on the relationship between SSBs and health (see above) highlights the value of cherry picking *inaccessible sources* (the report was not publicly available at the time of writing and had never been made publicly available via either the International Tax and Investment Center or Oxford Economics websites since publication), which compromises evidence appraisal.

The above examples illustrate a relatively uncomplicated, binary combination of agnogenic techniques. In practice, however, cherry-picking was combined in more subtle and complex ways with other techniques to amplify the significance of the relatively limited evidence-base challenging the effectiveness of SSB taxation (*evidential landscaping* – see Table 1). This is illustrated by Coca-Cola’s (and BEVSA’s) use of two studies by Fletcher, Frisvold and Tefft [97, 98] outlined in Table 4 below.

The first quote from the extract (A1) is taken from their 2010 examination of the effects of soft drink taxes in the US on child and adolescent soft drink consumption, substitution patterns, and weight outcomes. In the original the quoted text summarises the study’s results, but is used in Coca-Cola’s submission to imply that “several studies” had been reviewed (*selective quotation* and *illicit generalisation*) [90]. Moreover, the relevance of Fletcher, *at al*’s 2010 study to South Africa’s proposed SSB tax is unclear given that, historically, US tax rates have been significantly lower than that proposed by the South African Treasury. Fletcher, et al explicitly caution against extrapolating their results to large increases in tax rates (see A2 in Table 4), but this important qualification to their main finding, which immediately follows the extracted quote, is omitted (*omission of qualifying information*, see also *strategic ignorance* below).

The second quote is taken from a subsequent 2015 study by Fletcher, et al which specifically seeks to address the weaknesses noted in their earlier study by examining the weight trajectory of Ohio and Arkansas residents following large soda tax increases compared with individuals in other control states [98]. The study is well-designed and the quote fairly reflects the authors’ thoughts on the implications of their findings, but, once again, is limited to the study’s results rather than the “several studies” noted in the preceding sentence (*selective quotation* and *illicit generalisation*). In short, the agnogenic potential of cherry-picking, at least in this context, does not reside solely in which evidence is selected and which is ignored, although this is significant. Equally important is how cherry-picked evidence is subsequently dissembled, stripped of its context and qualifications, stitched back together and reframed.

#### Strategic ignorance

This inventive, unscientific use of research works to side-step a balanced consideration of the weight of evidence exploring the effects of SSB taxation on weight gain and obesity [94] and is symptomatic of an otherwise insoluble dilemma facing corporate actors: how to leverage the legitimacy of peer-review to support a strong dystopic position, where evidence is either emerging and uncertain, or simply contradicts their favoured claim [94]. In practice, *strategic ignorance* (Table 1) is key to resolving this dilemma. McGoey defines *strategic ignorance* as the “deliberate insulation from unsettling information” [28]. Whilst this broad definition has the advantage of highlighting corporate actors’ systematic tendency to ignore inconvenient evidence, it overlaps with *evidential landscaping* and fails to demarcate different modes of evidential exclusion. We use it in this context to describe the mixed practice of overlooking findings and evidence-backed observations in *cited* sources that work against dystopic claims-making. It is clearly apparent in *the omission of qualifying information* (see the discussion of Fletcher, et al immediately above), but also appears elemental in the industry’s efforts to piece together a coherent narrative (through observational selection/cherry-picking) from a body of knowledge largely unsympathetic to their dystopic narrative.

Coca-Cola and BEVSA, for example, ignored Malik and Hu’s (see above in relation to *The Tweezer’s method*) observation that “a majority of but not all systematic reviews had reported positive associations between SSB and weight gain or risk of overweight or obesity” [93], which undermined their efforts to raise doubts about the impact of SSBs on weight gain. Both corporate actors also overlooked their observation that SSBs had “been identified as a suitable target for public health interventions” because they “provide [d] “empty” calories and almost no nutritional value” [93], which countered their complaints that SSBs had being unfairly singled out for policy intervention. Equally, both submissions focused exclusively on the relationship between SSBs, weight gain, and obesity, ignoring strong evidence associating SSB consumption with elevated risk of type 2 diabetes [51, 102] even though this was explored at length by Malik and Hu. In fact, no mention is made of diabetes in either submission. Coca-Cola and BEVSA also ignored well-supported observations by Mozaffarian, et al. [103] (used in their submissions to highlight that other food categories had a stronger association with weight gain than SSBs) concerning the long-term effects of modest increases in weight over time, which worked against their efforts to highlight the minimal effect the SSB tax would have on health incomes because of the ostensibly small impact it was anticipated to have on daily average energy intake (Table 5). Perhaps the most egregious example concerned Coca-Cola’s (and BEVSA’s) claim that “consumers typically substitute [d] SSBs with other Calorie dense products”. This ignored assumptions made by Oxford Economics in its 2016 report that “research suggests negligible consumer switching into other beverages and foods following the rise in the price of SSBs” [58], which worked to inflate estimates of industry supported job losses and reduced tax revenue and GDP (see *Hyperbolic Accounting* below).

**Table 5:** Strategic Ignorance

**Text from Coca-Cola** [70] “Even by Treasury estimates, there will be very little impact, if any. Research cited by the Treasury in its policy paper finds that, in the central case, the proposed SSB tax will lower average energy consumption by only 36 kJ (8.6 Calories) per day (0.3%), equivalent to less than a quarter of an apple.” **Text from Mozaffarian, et al, 2011** [103] “Average long-term weight gain in nonobese populations is gradual — in the cohorts we studied, about 0.8 lb. (36 g) per year — but accumulated over time, even modest increases in weight have implications for long-term adiposity-related metabolic dysfunction, diabetes, cardiovascular disease, and cancer [104–107].”	

This technique also extended to claims concerning the economic impact of the policy. AmCham SA, for instance, used the 2013 Oxford Economics report [73] to support the claim that cross-border shopping had been “blamed for the loss of 1,300 jobs” in Denmark (see *Source Laundering* above) [71]. Immediately below it cited the Ecorys report to support a separate point outlining the negative effects of food taxes; ignoring Ecorys’ finding that the 30% increase in cross-border shopping claimed by industry stakeholders “was *not* confirmed in the Danish case study” [79] (see *Hyperbolic Accounting* below).

### Hyperbolic accounting

*Hyperbolic accounting* encompassed a range of interdependent techniques, which cumulatively worked to exaggerate the impact of SSB taxation on jobs, public revenue generation, and gross domestic product (GDP). Estimates of these impacts drew primarily on an economic impact analysis of the soft drinks industry summarised in Oxford Economics’ 2016 Report, whose own estimates were derived by summating the policy’s direct (economic activity supported by the core soft drinks industry), indirect (economic activity generated by the core industry’s supply chain, resulting from the procurement of domestically produced goods and services), induced (the wider economic effects of employees of the core soft drinks industry and its supply chain spending their earnings) and distribution (formal and informal retail, including spazas) impacts [58].

Some components of hyperbolic accounting, which we refer to as *acalculiac rounding-up* and *double-counting*, rested on simple misrepresentations of Oxford Economics’ estimates of impacts (*simple misstatements of key findings*). Both BEVSA and Coca-Cola, for example, claimed that the 2016 Oxford Economics report had estimated that the tax could lead to 62,000–72,000 lost jobs [70, 72] when it had, in fact, reported a range of between 60,600 and 70,700 potential job losses [58]. Likewise, after outlining Oxford Economics’ estimates for job losses, Coca-Cola and BEVSA noted that this could lead to the closure of between 8000 and 13,000 small retail outlets, based on each spaza employing 2 people and projected job losses of between 16,000 and 26,000 based on figures generated by Oxford Economics that included both spaza and wider local and traditional trade [70, 72]. However, Oxford Economics had in fact estimated that soft drinks sales could support between 13,400 and 23,500 fewer jobs in spaza stores following the tax, explicitly warning against adding spaza and local and traditional jobs together due to the risk of overlap between datasets and double counting [58].

Other examples were more artful and rested on subtle differences between how estimates were presented in Oxford Economics’ 2016 report and industry submissions. As indicated above, Oxford Economics’ projections of job losses and reductions in public revenue and GDP were the outcome of an industry-focused economic impact assessment. In contrast to policy-focused impact assessments, where a dynamic, whole-of-economy approach is taken to modelling the widest conceivable range of direct and indirect policy impacts, this takes a relatively static approach to modelling impacts proximate to the industry. Estimates were, therefore, limited to numerically describing the policy’s effect on the *industry’s* contribution to employment, government revenue, and GDP and did not take account of how the tax would displace economic activity to other parts of the economy or improve productivity [108]. As such, estimates of the effects of phenomena such as redeployed consumer spending, which offset the more proximate impacts of the tax [109], were not calculated. Oxford Economics was explicit on this point, noting, amongst other things, that calculations were on a “gross basis” and, therefore, did not “account for redeployment of spending by consumers outside of the soft drinks industry” [58] (see also Table 6, A1-A3). By contrast, BEVSA and Coca-Cola presented Oxford Economics’ estimates as conditional *policy* impacts - effects that would materialise provided its “least severe set of assumptions” held true (Table 6, B1-B3, BEVSA only). The natural inference to be drawn from quantifying the social costs of job losses (Table 6, B1), or referring to the “net impact on the fiscus” (Table 6, B2) or to providing categorical projections of reductions in GDP (Table 6, B3), was that Oxford Economics had taken a more comprehensive approach to modelling impacts (*false attribution of focus*) and that its estimates related to how the *policy* would affect jobs and gross value added across the economy, and, therefore, net employment, revenue generation and GDP.

**Table 6:** Conflating Industry-Specific and Economy-Wide Effects

**A1) Text from Oxford Economics (2016) on Jobs** [58] “The impact of the SSB tax on employment in spaza stores is based on the revenue impact of the tax estimated for local and traditional stores. This suggests that revenue from soft drinks sales could fall by around 22% in spaza stores due to the SSB tax. On that basis, *soft drinks sales could support* between 13,400 and 23,500 fewer jobs in spaza stores following the tax, depending on whether jobs are estimated based on soft drinks’ share of revenue or margins.” **A2) Text from Oxford Economics (2016) on Public Revenue Generation** [58] “We estimate that this reduction in economic activity could reduce *the industry’s contribution* to tax revenues by R3.1 billion, including VAT due to lower sales volumes.” **A3) Text from Oxford Economics (2016) on GDP** [58] “Once the multiplier impacts are considered, *the contribution of the core soft drinks industry* to South Africa GDP could decline by R14 billion.” **B1) Text from BEVSA (2016) on Job Losses** [72] “The report from Oxford Economics (see Economic impact methodology sidebar) estimates that the proposed SSB tax could result in the loss of 62,000–72,000 existing jobs (3400 direct, 25,200 upstream, and 15,400 induced job losses; combined with 19,000–29,000 downstream job losses). The industry estimates that this will prevent the creation of 18,000–28,000 planned new jobs over the next three years. The tax could force the closure of 8000–13,000 small retail outlets and spaza shops … ..Standard approaches put the social cost of the increase in mortality, due to the job losses caused by the SSB tax, at more than R1 billion. This is in addition to the other social effects of unemployment, such as increased violent crime.” **B2) Text from BEVSA (2016) on Public Revenue Generation** [72] “The report by Oxford Economics estimates that job losses and lower industry profits could reduce Government revenues from its existing taxes by at least R3.1 billion per annum. The Government could see personal income taxes fall by R1.3 billion, corporate income taxes fall by R1.1 billion, and VAT reduced by R0.8 billion. In addition, the tax would, through its impact on unemployment, result in increased UIF payments of approximately R0.7 billion, as well as additional (unquantified) costs to the fiscus from secondary socio-economic effects of unemployment. As a result, the net impact on the fiscus from the SSB tax could be 50% lower than expectations.” **B3) Text from BEVSA (2016) on GDP** [72] “Using the least severe set of assumptions, the effects described above could reduce South Africa’s GDP by R14 billion (R3.5 billion direct, R6.7 billion indirect, and R3.8 billion induced GDP contribution).”	

#### Syncopated estimation

We use *syncopated estimation* to describe Oxford Economics’ practice of skipping steps in economic modelling (Table 1). The technique illustrates the challenges in unscrambling hyperbolic accounting where industry actors enjoy a legitimate measure of discretion in making assumptions relevant to predicting future consequences. A mix of mutually reinforcing agnogenic practices were used to justify assumptions about how consumers would respond to SSB taxation, exploit gaps in the peer-reviewed research on cross-price elasticities (see below) and substitution effects, and ultimately provide a basis for modelling complements (see below) that inflated headline estimates and ignored substitutes that had deflationary effects.

In brief, estimates produced within economic impact assessments depend in large part on the type of assumptions made about future behaviour [110]: outwardly innocuous choices over what eventualities get modelled and how, can, potentially, have far-reaching effects on final estimates (relating, in this case, to industry supported employment, tax revenue, and GDP). Ideally, models should provide a spread of estimates representing different assumptions about how policies are likely to take effect. Assumptions should take account of the best available evidence and the range of assumptions modelled should reflect the degree of uncertainty within the evidence: the less developed or consistent the evidence, the stronger the case for models to consider a range of assumptions and the greater the range of modelled estimates. The primary uncertainty in predicting the impact of South Africa’s SSB tax on the soft drinks industry centred on how the expected increase in SSB prices might influence consumer demand for other products manufactured by the industry, such as bottled waters, flavoured and/or enhanced waters, ready-to-drink teas and coffees, and dairy-based beverages [72]. In microeconomic modelling, changes in consumer demand for products in response to a change in price of another product are measured by cross-price elasticities. Two products that are substitutes have a positive cross elasticity of demand (as the price of SSB rises, the demand for the other product also rises), whereas two products that complement one another have a negative cross elasticity of demand (as the price of SSB rises, the demand for the other product falls). Modelling fewer substituted products manufactured by the industry or purposively selecting complements would, therefore, serve to reduce the extent to which predicted consumer choices offset the decline in SSB sales, inflating the projected impact of the tax on industry supported employment, tax revenue, and GDP.

In practice, Oxford Economics modelled for fruit juice and diet drinks only [58]; using cross-price elasticities reported in a 2014 analysis by Manyema, et al [111] (hereafter just Manyema, et al), which drew on values calculated in a 2013 meta-analysis of studies covering the USA, France, Mexico and Brazil [112] (hereafter just Cabrera Escobar, et al). Cabrera Escobar, et al had reported a limited number of substitutes (fruit juice and milk), and a negative cross-price elasticity for diet drinks, which (in Oxford Economics’ modelling) offset the extent to which substitution to fruit juice moderated the predicted impact of reduced SBB consumption on industry revenue. Although drawing on the results of a meta-analysis suggested methodological rigour, relying on Cabrera Escobar, et al for cross-price elasticities effectively exploited fundamental differences in approaches to modelling health effects of SBB taxes where excluding product substitutions considered less harmful to health is not uncommon. Several studies reviewed by Cabrera Escobar, et al, for example, had estimated cross-price elasticities for other products, such as bottled water [48, 113, 114] and tea and coffee [48, 113] (see Additional file 1: Table S1). However, both these and cross-price elasticities for other beverages, such as milk, were omitted from their review as they contained “some nutritional value” and “none of them contain [ed] sugar added prior to packaging, so their relationship with obesity [was] not as direct as it is for SSBs” [112]. More to the point, Cabrera Escobar, et al represented a *passé source*, which although fairly reflecting the state of scientific knowledge when published had since been superseded by developments in the evidence-base. Research on product substitutes and complements is a fast-developing area. Only two studies [113, 115] reviewed by Cabrera Escobar, et al, for example, had estimated cross-price elasticities for diet drinks. Notwithstanding continuing gaps in cross-price elasticities for beverages produced by the industry, subsequent studies have reported positive values for diet drinks [90, 116] and bottled water [116] (see Additional file 1: Table S1). Given the undeveloped state of the literature in 2013 (the year of Cabrera Escobar, et al’s publication), ongoing inconsistencies in reported estimates for cross-price elasticities (Additional file 1: Table S1), and the fact that reported values vary geographically [80] and in response to different methods of estimation [48], it was incumbent on Oxford Economics to either generate their own estimates for cross-price elasticities from South African consumer panel data or to provide a range of estimates based on different values for cross-price elasticities that reflected the variation in the literature.

In the event, Oxford Economics produced categorical, rather than a range of, estimates and made several attendant observations that (outwardly) supported its exclusive reliance on Cabrera Escobar, et al as a source of cross-price elasticities. For example, it argued that using Manyema et al (and, by implication, Cabrera Escobar, et al) as a source of cross-price elasticities was necessary “to ensure that the key assumptions underpinning [its] work [were] consistent with those reported in the National Treasury’s SSB tax policy paper” [58]. This implied that the Treasury had used their findings to model projected impacts of the tax. In fact, the Treasury’s policy paper contained no detailed modelling. Manyema, et al was simply one of several studies cited to indicate the potentially positive effects of an SSB tax on health outcomes [56] and reference to the policy paper, as such, represented little more than a *faux source* or appeal to a *false authority*. Further, Oxford Economics claimed that Manyema et al. had reported that, “drinkers of SSBs [were] unlikely to switch to bottled water” and that “other studies [had] not found statistically robust evidence that people switch from SSBs to water when the price of SSBs increase” [58]. In fact, Manyema, et al make no reference to bottled water (*simple misstatement of study findings*). Moreover, whilst some studies had found no evidence of substitution to water (see Additional file 1: Table S1), many other studies (prior to 2016) had. This was reflected in Manyema, et al’s observation that, “other studies have shown that the demand for tea and coffee, as well as water goes up with SSB price increases”, which cited a 2013 study [90] published in the *British Medical Journal* in support that Oxford Economics had also cited to make a separate point (*strategic ignorance*).

#### Information asymmetries, inaccessible data and black box computation

Finally, the scope for *hyperbolic accounting* was enabled by reliance on privately held data in economic assessments (*inaccessible data*) and opaque, unverifiable steps in economic modelling (*black box computation*). Although not strictly agnogenic practices, both phenomena increase the obstacles involved in substantiating the bona fides of industry estimates of projected economic impacts and, as such, expand the opportunities for corporate agnogenesis. For example, Oxford Economics’ estimates of lost spaza jobs and store closures rested on the assumption that *post-tax* employment would fall in proportion to the decline in revenue generated by soft drink sales. Its projections were, therefore, tied to baseline estimates for both employment and store numbers and the proportion of spaza revenue (and profit margins) derived from SSB sales. In relation to the former, estimates for both employment and store numbers drew on unpublished, industry-funded research by PwC [117] and “consultation with industry” [58], which had the effect of increasing PwC’s original estimate of 150,000 “small business enterprises” and 300,000 workers to 180,000 stores, employing 360,000 people [58]. Likewise, revenue estimates were based on unpublished industry surveys, which suggested that approximately 17% of store turnover (30% of retail margin) was attributable to soft drink sales. On first examination, *black box computation* in Oxford Economics’ report appeared relatively trivial and was partly eclipsed by the inclusion of appendences that provided clear summaries of both its economic impact methodology and approach to estimating the impact of the SSB tax on the economic footprint of the soft drinks industry in appendices to its report. Nonetheless, various figures and values were effectively asserted without adequate explanations of their provenance which were key to Oxford Economics’ final estimates. For instance, Oxford Economics’ simply noted that its “modelling suggests that the core industry paid R1.8 billion in corporation tax and almost R1.1 billion in income tax payments”, without providing either the data underlying or method of its calculations. Equally, the report noted that, “[u] sing industry specific productivity estimates derived from Statistics South Africa data and published by Oxford Economics the core industry [was] estimated to support around 107,500 jobs indirectly and a further 66,500 via the induced impact channel” without outlining how they had been derived [58].

Finally, focusing exclusively on information asymmetries in respect of Oxford Economics’ estimates belies the true extent of the problem in corporate submissions. Some sources (e.g. Oxford Economics’ 2013 report, which was drawn on heavily by AmCham SA) referred to directly in corporate submissions to support other points, for example, were not publicly available either during the consultation period or at the time of writing. The same applied to other sources that underpinned claims in cited references (e.g the survey by the Danish Grocer’s Trade Organisation - see under Cryptic Referencing).

### Modelling corporate agnogenesis in the soft drinks industry

Figure 1 depicts the above findings and seeks to illustrate how the industry’s agnogenic techniques relate to one another.

Some techniques and practices identified in our data overlap with those outlined in Ulucanlar et al’s study of the tobacco industry: notably, the tweezers method, the conflation of absence of evidence with evidence of absence, and evidential landscaping [5]. The industry’s use of logical fallacies and its practice of cherry-picking also highlight commonalities with denialism [99, 118]. Further, some examples of cherry-picked evidence (see the discussion of Fletcher, et al under *Observational Selection* above) have the effect of “wholesale discounting of evidence” [5] also reported in Ulucanlar et al*’s* study.

In addition, the model outlines four qualities of corporate agnogenesis that illustrate its plasticity and the interdependence between different techniques and practices. First, different techniques can produce the same effect. Both the *tweezers method* and *false attribution of focus*, for example, were used to conflate *absence of evidence with evidence of absence*. Second, the same technique can be used to produce different effects. *Out-of-place citations*, for example, were used to support *illicit generalisations* and *misleading summaries*. Third, agnogenic techniques can operate as a series of steps in a process or a *chain of agnogenesis*. For instance, key qualifying information was omitted from studies which worked to misrepresent their focus and objectives and, ultimately, provided a platform to present contradictory findings on the extent to which food taxes led to improvements in health (an absence of consistent evidence) as evidence that the research had found no improvements (evidence of absence). Fourth, agnogenic techniques can also combine in more complex ways. Coca-Cola’s and BEVSA’s practice of cherry-picking studies to support their preferred theory of substitution effects, for example, rested on an *illicit generalisation* and relied heavily on *selective quoting* and the *omission of qualifying information* of the selected studies, as well as *strategic ignorance* of the findings and observations in studies relied on elsewhere in their submissions. In this respect, agnogenesis is produced via a mutually reinforcing *network of agnogenic techniques* that produce superficially coherent descriptions of evidence that support the industry’s overarching dystopic narrative.

Finally, account needs to be taken of the different roles that agnogenic practices perform in corporate evidence claims. Some, such as *selective quotations*, for example, constitute classic agnogenic techniques in so far as they misrepresent the underlying evidence. Others, however, such as *cryptic references*, *inaccessible data and black box computation* perform a more ancillary role: not misleading in themselves, but, nonetheless, potentially instrumental to more direct agnogenic practices in so far as they work to obstruct evidence appraisal. Equally, *strategic ignorance* is neither strictly misleading (although it may be) nor obstructive of evidence appraisal. However, it represents a necessary strategy in building agnogenic narratives where the weight of evidence suggests a policy is broadly likely to work as intended.

## Discussion

Corporate agnogenesis represents a major problem for health actors and the general population. Addressing the global non-communicable disease epidemic requires fundamental changes in markets: what products are sold, at what price, how and to whom [119]. It sets publics and health professionals against transnational corporations in what are partly ideational [120] and partly evidence-based policy conflicts that must take account of what works and at what costs. In this respect, agnogenic practices need to be understood both as political techniques in their own right and as components of other political techniques [121–123], such as direct lobbying and constituency building, where the communication of evidence-based information is instrumental to framing issues [69].

This political potential of corporate agnogenesis has been strengthened by the emergence of new forms of policy-making governance, which draw heavily on the US cost-benefit approach to policy formation. These new forms of governance have elevated the importance of evidence in areas of policy-making in which corporate and public interests clash and enhanced the effective political power of economics which has created a receptive milieu for industry commissioned economic impact analyses that translate diverse and complex processes into a single figure with the sense of precision and neutrality widely accorded to numbers [124]. Providing support for both health and economic-related claims, engaging with the peer-reviewed literature, and presenting economic estimates with the appearance of a sound theoretical basis, establishes a right to be heard and taken seriously. Corporate agnogenesis then goes on to exploit the uncertainties inherent in both scientific norms and practices and economic modelling that this right of policy engagement affords.

These uncertainties highlight the structural vulnerability of modern modes of evidence-based policy-making to corporate agnogenesis. Scientific uncertainty arises in part because new evidence is constantly emerging and because new methods are regularly developed to gather and analyse evidence. No scientific claim is entirely free from evidential challenge [125, 126]. Corporations also, in effect, leverage the culture of criticism that scientists seek to cultivate, which involves pointing out where other scientists have overstated their findings, or missed important things, and developing alternative explanations of the evidence [6, 127–130]. In the present case, these characteristics of scientific uncertainty are exemplified by the research on cross-price elasticities, where relatively large variations in values persist and different methodological approaches have produced conflicting findings on substitution effects. Both were exploited in industry submissions. By contrast, the agnogenic risks inherent in industry economic modelling arise from the conceit underlying estimates that appear to render the future knowable and calculable. In practice, each assumption and step in the modelling process provides a further opportunity to inflate the projected costs of public health policies. The potential agnogenic effect is compounded by a lack of clarity in how data sources have been produced, and by mishandling inconsistencies and gaps in the scientific literature. The industry’s production of categorical estimates for economic impacts, as opposed to a range, simply reflects the political (and therefore commercial) peril in embracing uncertainty, which, inevitably, would lead to less conclusive outputs and lower estimates of effects. Consequently, estimates of impacts are precise, but not necessarily accurate, a subtle artefact of cumulative overstatement that produces precision from imprecision. The risk to evidence informed policy-making is that headline estimates, rather than the questionable and indiscernible assumptions that underpin them have the greater salience, and mnemonic potential.

Structural weaknesses inherent in contemporary forms of evidence-based policy-making are exacerbated by diagnostic problems in unpicking corporate agnogenic techniques. On first inspection, these appear to be softened by broad similarities between agnogenic techniques identified in studies of corporations’ use of evidence in different sectors, which is providing an emerging inventory of sharp evidential practices [5]. Despite this, the way in which identical practices manifest themselves in different evidential and policy contexts is necessarily unique and not only requires careful appraisal of the specific evidence upon which they profess to be based, but also knowledge of the broader evidential context in which they are set. In addition, while singular, agnogenic techniques do not work to create doubt in isolation, but rather work in chains and networks of agnogenic logic. This layered quality of agnogenesis produces an ostensibly coherent, evidence-based analysis, which, when combined with other techniques, such as confounding references, that directly inhibit evidence appraisal, work to intensify the difficulties of detection. Finally, these difficulties are compounded by agnogenic techniques that use evidence widely regarded as high quality. Observational selection (cherry-picking), selective quotations, the false attribution of focus, and the tweezers method, for instance, typically involved peer-reviewed studies. Evaluating the strength and validity of the evidence in industry submissions, as such, cannot be simply divined by examining the quality of evidence cited or funding sources which by-passes the conceit underlying agnogenesis: its mimicry of the core commitment to evidence-based reasoning within scientific norms and practices.

## Conclusions

The policy implications of our findings need to be set against the spread internationally of regulatory impact assessments and mandatory public consultations through Better Regulation/Good Governance agenda [131] (and their equivalents) and contemporary trade and investment agreements [3]. By prescribing a right to submit evidence and embedding cost-benefit analysis within policy-making, these formalise opportunities for corporate agnogenesis and the political potential of industry-funded economic modelling. That these policy instruments work in the interests of corporate actors is consistent with calls from BEVSA and Coca-Cola for the South African government to undertake a full socio-economic impact assessment of the policy, in consultation with the industry [70, 72].

Further, our findings not only highlight the value of improving the transparency and scrutiny of regulatory impact assessments and consultations in health policy-making, but also other modes of industry political activity. In the present context, for example, the findings of industry commissioned research, including the 2016 Oxford Economics report, for example, were cited in stakeholder workshops organised by the National Treasury [132]. In addition, the fact that some of the practices and techniques outlined above have been used in various, policy-related contexts (by, for example, actors linked to the tobacco [5, 118, 133], alcohol [34, 39], fossil fuel [31, 99, 118, 130, 134], chemical [37] and agrochemical industries [37]) highlights both the relevance of our work to other policy fields and the importance of ensuring full transparency across all areas of policy-making where corporate interests run-up against broader public interests. Full transparency would involve publication of all industry submissions to consultations and verbatim transcripts of workshops, correspondence and meetings between industry actors and officials, and should be formalised within the context of “policy footprints”. These represent a real-time record of lobbyists’ influence on policy, which mandate disclosure of all contacts and correspondence with officials, minutes of meetings, and any supporting materials relied on or provided by lobbyists in the course of policy development [135, 136]. Comprehensive policy footprints represent one of several reforms necessary to meet the recommendation of the recent Lancet Commission on Obesity, Undernutrition and Climate [137] for the introduction of an international agreement to address conflicts of interest in food policy. However, transparency alone is unlikely to be enough to manage the effects of corporate agnogenesis in health policy, given the difficulties in unpicking how it takes effect. In addition, efforts need to be made to enhance appraisal of industry use of evidence. Ideally, there should be a presumption in favour of in-depth critical appraisal, organised and financially supported by national governments. Beyond this, there is a strong case for closer transnational collaboration between civil society actors and academics that centres on producing real-time appraisals of companies’ use of evidence in both public consultations and other contexts in which they provide information to policy actors and the public.

Given the policy risks associated with corporate agnogenesis there is a need for further, in-depth research on corporations’ use of evidence in different policy areas relevant to public health (e.g. climate change, environmental health, occupational health, alcohol, agrochemicals, and gambling), as well as in respect of different polices relevant to diet-related diseases (e.g. restrictions on marketing to children). More generally, our findings point to the importance of further research on the political-psychology of corporate agnogenesis. The obvious explanation for corporate agnogenesis is that it represents a necessary protective strategy for business actors where the evidence-base necessary to contest commercially prejudicial policies is weak or unhelpful. However, submissions were characterised by a form of *kettle logic*, a term coined to describe the use of multiple, contradictory arguments to support a single point [138], which reflected the legalistic style of corporate submissions where efforts to raise every conceivable objection to a policy and the evidence supporting it led to industry actors taking positions that appeared credible when viewed in isolation, but which were, in fact, confused and contradictory when viewed collectively. In its most basic form, this involved industry actors claiming that SSB taxation would not generate the revenue projected, not affect consumption because consumers would merely accept the tax, and lead to catastrophic job losses and business closures [71]. In our view, this elemental flaw in industry submissions merits further examination of the thinking underlying discrete cases of corporate agnogenesis, which combines the conceptual tools of political economy and organisational psychology. Finally, further research is required to explore the effects of corporate agnogenesis on the perceptions of policy actors and publics.

## Additional file

Additional file 1: **Table S1.** Peer reviewed research articles on SSB taxes (published prior to 2016) containing analyses based on calculated cross-price elasticities. (DOCX 16 kb)

## Data Availability

Not applicable.

## Abbreviations

AmCham SA: American Chamber of Commerce South Africa
BEVSA: Beverage Association of South Africa
FAO: Food and Agriculture Organization of the United Nations
GDP: Gross domestic product
SSB: Sugar-sweetened beverages
