# Supplementary material for: Corporations’ use and misuse of evidence to influence health policy: a case study of sugar-sweetened beverage taxation
Source: Global Health. 2019 Sep 25;15:56. doi: 10.1186/s12992-019-0495-5 (PMC6760066; doi:10.1186/s12992-019-0495-5)
Supplement: Supplementary file 1 — Table S1. Peer reviewed research articles on SSB taxes (published prior to 2016) containing analyses based on calculated cross-price elasticities. (DOCX 16 kb) [file 12992_2019_495_MOESM1_ESM.docx]

**S1 Table Peer reviewed research articles on SSB taxes (published prior to 2016) containing analyses based on calculated cross-price elasticities**

| Country | Study | Data Source | Diet soft drinks | Full-fat milk | Low-fat milk | Fruit drinks | Fruit juices | Bottled water | Coffee | Tea | Diet flavoured or enhanced water | Sports energy drinks |
| --- | --- | --- | --- | --- | --- | --- | --- | --- | --- | --- | --- | --- |
| Mexico | Barquera S, Hernandez-Barrera L, Tolentino ML, Espinosa J, Ng SW, Rivera JA, Popkin BM: Energy intake from beverages is increasing among Mexican adolescents and adults. J Nutr 2008, 138(12):2454–2461. | Survey Data | Not modelled | Positive (0.052) | Not modelled | Not modelled | Negative (-0.112) | Positive (0.023) | Not modelled | Not modelled | Not modelled | Not modelled |
| USA | Finkelstein EA, Zhen C, Nonnemaker J, Todd JE: Impact of targeted beverage taxes on higher-and lower-income households. Arch Intern Med 2010, 170(22):2028–2034. | Nielsen’s National Consumer Panel (Homescan) | Not reported | Not reported | Not reported | Not reported | Not reported | Not modelled | Not modelled | Not modelled | Not modelled | Not reported |
| USA | Smith, T.A., Lin, B.H., Lee, J.Y., Taxing Caloric Sweetened Beverages: Potential Effects on Beverage Consumption, Calorie Intake, and Obesity. Economic Research Report #100. 2010. USDA, Washington, DC. | Nielsen’s National Consumer Panel (Homescan) / Suvey Data | Negative (-0.46) | Positive (0.2) | Positive (skim milk) (0.2) / (low-fact milk) (0.11) | Not reported | Positive (0.56) | Positive (0.75) | Negative (coffee and tea considered together) (-0.38) | Negative (coffee and tea considered together) (-0.38) | Not reported | Not reported |
|  | Duffey KJ, Gordon-Larsen P, Shikany JM, Guilkey D, Jacobs DR, Popkin BM. Food price and diet and health outcomes: 20 years of the CARDIA study. Arch Intern Med. 2010;170(5):420–426 | Survey Data | Not modelled | Positive (value not reported) | Not modelled | Not modelled | Not modelled | Not modelled | Not modelled | Not modelled | Not modelled | Not modelled |
| USA | Fletcher MJ, Frisvold DE, Tefft N: Can soft drink taxes reduce population weight? Contemp Econ Policy 2010, 28(1):23–35. | Survey Data | Not reported | Not reported | Not reported | Not reported | Not reported | Not reported | Not reported | Not reported | Not reported | Not reported |
| USA | Fletcher JM, Frisvold DE, Tefft N: The effects of soft drink taxes on child and adolescent consumption and weight outcomes. J Public Econ 2010, 94(11):967–974. | Survey Data | Not reported | Positive (value not reported) | Not modelled | Positive (value not reported) | Negative (value not reported) | Not modelled | Not modelled | Not modelled | Not modelled | Included in modelled tax |
| USA | Lin BH, Smith TA, Lee YJ: Measuring weight outcomes for obesity intervention strategies: the case of a sugar-sweetened beverage tax. Econ Hum Biol 2011, 9:329–341. **low income population** | Nielsen’s National Consumer Panel (Homescan) | Negative (-0.23) | Positive (0.242) | Negative (-0.25) | Included in modelled tax | Positive (0.473) | Negative (-0.027) | Negative (coffee and tea considered together) (-0.408) | Negative (coffee and tea considered together) (-0.408) | Not modelled | Included in modelled tax |
| USA | Lin BH, Smith TA, Lee YJ: Measuring weight outcomes for obesity intervention strategies: the case of a sugar-sweetened beverage tax. Econ Hum Biol 2011, 9:329–341. **high income population** | Nielsen’s National Consumer Panel (Homescan) | Negative (-0.591) | Negative (-0.054) | Positive (0.344) | Included in modelled tax | Positive (0.529) | Positive (1.005) | Negative (coffee and tea considered together) (-0.185) | Negative (coffee and tea considered together) (-0.185) | Not modelled | Included in modelled tax |
| France | Bonnet C, Requillart V: Does the EU sugar policy reform increase added sugar consumption? An empirical evidence on the soft drink market. Health Econ 2011, 20(9):1012–1024. | Consumer Panel Data (TNS WordPanel) | Not reported | No modelled | Not modelled | Not modelled | Not modelled | Not modelled | Not modelled | Not modelled | Not modelled | Not reported |
| USA | Zhen C, Wohlgenant MK, Karns S, et al. Habit formation and demand for sugar-sweetened beverages. Am J Agric Econ. 2011;93:175–193. **low income population** | Nielsen’s National Consumer Panel (Homescan) | Negative (value not reported) | Positive (value not reported) | Positive (value not reported) | Included in modelled tax | Positive (value not reported) | Not modelled | Not modelled | Not modelled | Not modelled | Included in modelled tax |
| USA | Zhen C, Wohlgenant MK, Karns S, et al. Habit formation and demand for sugar-sweetened beverages. Am J Agric Econ. 2011;93:175–193. **high income population** | Nielsen’s National Consumer Panel (Homescan) | Negative (value not reported) | Positive (value not reported) | Positive (value not reported) | Included in modelled tax | Positive (value not reported) | Not modelled | Not modelled | Not modelled | Not modelled | Included in modelled tax |
| USA | Dharmasena S, Capps O: Intended and unintended consequences of a proposed national tax on sugar-sweetened beverages to combat the U.S. obesity problem. Health Econ 2012, 21(6):669–694. | Nielsen’s National Consumer Panel (Homescan) | Negative (-0.86) | Positive (0.19) | Positive (0.64) | Negative (-0.37) | Positive (1.28) | Positive (0.03) | Positive (0.70) | Positive (0.36) | Not modelled |  |
| Brazil | Claro RM, Levy RB, Popkin BM, Monteiro CA: Sugar-sweetened beverage taxes in Brazil. Am J Public Health 2012, 102(1):178–183. | Survey Data | Not reported | Not reported | Not reported | Not reported | Not reported | Not reported | Not reported | Not reported | Not reported | Not reported |
| Brazil | Claro RM, Levy RB, Popkin BM, Monteiro CA: Sugar-sweetened beverage taxes in Brazil. Am J Public Health 2012, 102(1):178–183. | Survey Data | Not reported | Not reported | Not reported | Not reported | Not reported | Not reported | Not reported | Not reported | Not reported | Not reported |
| USA | Finkelstein EA, Zhen C, Bilger M, Nonnemaker J, Farooqui AM, Todd JE: Implications of a sugar-sweetened beverage (SSB) tax when substitutions to non-beverage items are considered. J Health Econ 2013, 32:219–239. | Nielsen's National Consumer Panel (Homescan) | Negative (value not reported) | Negative (value not reported) | Positive (value not reported) | Included in modelled tax | Positive (value not reported) | Not modelled | Not modelled | Not modelled | Not modelled | Included in modelled tax |
| UK | Briggs, A.D.M., et al., Overall and income specific effect on prevalence of overweight and obesity of 20% sugar sweetened drink tax in UK: econometric and comparative risk assessment modelling study. BMJ : British Medical Journal, 2013. 347. | Survey Data | Positive (excludes concentrated diet soft drinks) 0.17 | Positive (milk only) (0.157) | Positive (milk only) (0.16) | Included in modelled tax | Positive 0.13 | Not modelled | Positive (tea and coffee together) 0.17 | Positive (tea and coffee together) 0.17 | Not modelled | Included in modelled tax |
| Australia (Victoria) | Sharma, A., et al., The Effects of Taxing Sugar Sweetened Beverages across Different Income Groups. Health Economics, 2014. 23(9): p. 1159-1184. | Nielsen's Consumer Panel Data (Homescan) | Positive (0.16) | Positive (0.46) | Positive (0.12) | Negative (-0.62) | Positive (0.18) | Positive (0.37) | Negative (-0.89) | Negative (-0.89) | Not modelled | Included in modelled tax |
| USA | Zhen, C., et al., Predicting the Effects of Sugar-Sweetened Beverage Taxes on Food and Beverage Demand in a Large Demand System. American Journal of Agricultural Economics, 2014. 96(1): p. 1-25. | Nielsen’s National Consumer Panel (Homescan) | No change | Positive (0.05) | Negative (-0.18) | Positive (0.35) | Negative (-0.29) | Negative (-0.20) | Not modelled | Not modelled | Not modelled | Positive (0.26) |
| India | Basu, S., et al., Averting Obesity and Type 2 Diabetes in India through Sugar-Sweetened Beverage Taxation: An Economic-Epidemiologic Modeling Study. PLOS Medicine, 2014. 11(1): p. e1001582. | Survey Data | Not modelled | Positive (milk only) 0.049 | Not modelled | Not modelled | Positive (0.31) | Not modelled | No effect detected (0.0041) | Positive (0.13) | Not modelled | Not modelled |
| UK | Tiffin, R., A. Kehlbacher, and M. Salois, The effects of a soft drink tax in the UK. Health Econ, 2015. 24(5): p. 583-600. | KANTAR WorldPanel and Survey Data | Negative for high and moderate consumers of SSBs, but positive for low consumes (values not reported) | Not modelled | Not modelled | Included in modelled tax | Negative | Negative for high and moderate consumers of SSBs, but positive for low consumers (values not reported) | Not modelled | Not modelled | Not modelled | Negative for high consumers SSBs, but positive for moderate and low consumes (values not reported) |
| Mexcio | Colchero MA, Salgado JC, Unar-Munguia M, Hernandez-Avila M, Rivera-Dommarco JA. Price elasticity of the demand for sugar sweetened beverages and soft drinks in Mexico. Economics and human biology. 2015;19:129-37. | Survey data | Not modelled | Positive (milk only) (0.11) | Positive (milk only) (0.11) | Explored as "other SSBs" and not disaggregated (negative, -0.21) | Explored as "other SSBs" and not disaggregated (negative, -0.21) | Explored as "natural and mineral water" (positive, 0.07) | Not modelled | Not modelled | Explored as "other SSBs" and not disaggregated (negative, -0.21) | Explored as "other SSBs" and not disaggregated (negative, -0.21) |
